# Hymenoptera Allergy Diagnosis through Their Presence on Human Food

**DOI:** 10.3390/toxins15120680

**Published:** 2023-12-01

**Authors:** Arantza Vega-Castro, Leopoldo Castro, Francisco Carballada, Teresa Alfaya, Lluís Marquès, Berta Ruíz-León

**Affiliations:** 1 Allergy Service, University Hospital of Guadalajara, 19002 Guadalajara, Spain; 2 IDISCAM (Instituto de Investigación de Castilla la Mancha), 45071 Toledo, Spain; 3 IES Vega del Turia, 44002 Teruel, Spain; rhynchium@gmail.com; 4 Allergy Service, HULA (Hospital Universitario *Lucus Augusti*), 27003 Lugo, Spain; francisco.carballada.gonzalez@sergas.es; 5 Allergy Service, Hospital Universitario Fundación Alcorcón, 28922 Madrid, Spain; teresa.alfaya@salud.madrid.org; 6 Allergy Service, Hospital Universitari Arnau de Vilanova, 25198 Lleida, Spain; lmarques.lleida.ics@gencat.cat; 7 Instituto de Investigación Biomédica de Lleida, 25198 Lleida, Spain; 8 Allergy Service, Hospital Universitario Reina Sofía, 14004 Córdoba, Spain; rulebe@gmail.com; 9 Instituto Maimónides de Investigación Biomédica, 14004 Córdoba, Spain

**Keywords:** Hymenoptera, *Vespula*, *Polistes*, *Vespa*, venom allergy, diagnosis, wasp foraging, Hymenoptera sting, human food

## Abstract

Venom immunotherapy (VIT) protects up to 98% of treated Hymenoptera allergy patients from reactions with new stings. A correct diagnosis with the identification of the venom causing the allergic reaction is essential to implementing it. The knowledge of the Hymenoptera foraging habits when the sting takes place in a food environment would allow the culprit insect to be known. Images of Hymenoptera occurring in environments where there was human food were recorded in Spain, including the date of the image, the place description and its geolocation. The insects’ genus and species were identified by an entomologist. Results: One hundred and fifty-five images depicting 71 insects were analyzed. The identified insects were *Vespula* (56), *Vespa* (7), *Polistes* (4), *Cerceris* (2), *Bombus* (1) and *Apis* (1). Most (97.1%) of the images were obtained in summer and early autumn, outdoors in terraces (64%). Meat was the food associated with 47.9% of the images. In protein-rich foods, *Vespula* was found in 89%. Conclusions: *Vespula* was the main Hymenoptera associated with food environments in our country (78.87%), and in most of the cases (71%), the food involved is a source of protein, such as meat or seafood. In that environment, the probability that the insect is a *Vespula* would be 89%.

## 1. Introduction

Allergy to Hymenoptera venom can cause serious reactions that endanger the patient’s life. There are abundant descriptions of the efficacy of immunotherapy in such cases, with protection for new stings reaching up to 98% of treated patients [1]. 

A key point in the efficacy of immunotherapy is diagnosis, which leads to the correct identification of the venom causing the allergic reaction. In most cases, the patient is unable to accurately identify the stinging insect; on other occasions, double positivity to Hymenoptera, especially in the case of vespids, makes it necessary to use additional diagnostic methods to find out which vespid caused the reaction [2].

The environment in which the stinging incident takes place can shed light on the insect causing the allergic reaction since the behavior of the species of *Vespula* (Thomson, 1869), *Vespa* (Linnaeus, 1758), and *Polistes* (Latreille, 1802) (Hymenoptera: Vespidae) differs as to their habits and food sources [3,4]. According to the literature, *Vespula* will use processed and other foods consumed by man, while *Polistes* does not. Adult vespids feed on carbohydrates that they mainly obtain from flower nectar. *Vespula* and *Vespa* also eat ripe fruit and can therefore enter orchards, vineyards and other fruit yards, as well as open market stalls, picnic areas, houses, etc. *Polistes* species are scarce in fruit orchards, except possibly on fig trees, and do not look for food at market stalls, shops or picnic areas.

On the other hand, the food that workers and queens collect for their larvae mostly consists of proteins that come from the tissues of the invertebrates, mainly insects, that they catch. Some species of *Vespula* and *Vespa* will also use the meat of dead animals, both invertebrates and vertebrates; some of it is proteic food, either raw or cooked (meat, sausages, fish, mollusks, etc.), destined for human consumption [5,6,7,8,9].

If the food habits of the various vespid species are known, it may be possible to know, in certain environments, which insect was responsible for the sting. We suggest that the sting received in a human food environment will almost certainly have been caused by Hymenoptera of the genera *Vespula* or *Vespa*. This would provide, in the case of some patients, an important tool for diagnosis that would do away with the need for more complex and costly testing.

In order to confirm our hypothesis, we have conducted a study that identifies the Hymenoptera species found in human food environments.

## 2. Results

The study was carried out from September 2017 to October 2022. One hundred and sixty-four images were received. Of those, 150 photographs and five videos depicting 71 insects met the given requirements. The remaining nine images were discarded, either because the quality was poor and insufficient for a reliable identification of the insect or because they were not linked to a food environment. 

### 2.1. Identified Insects: Places and Time of the Year

**Figure 1:**
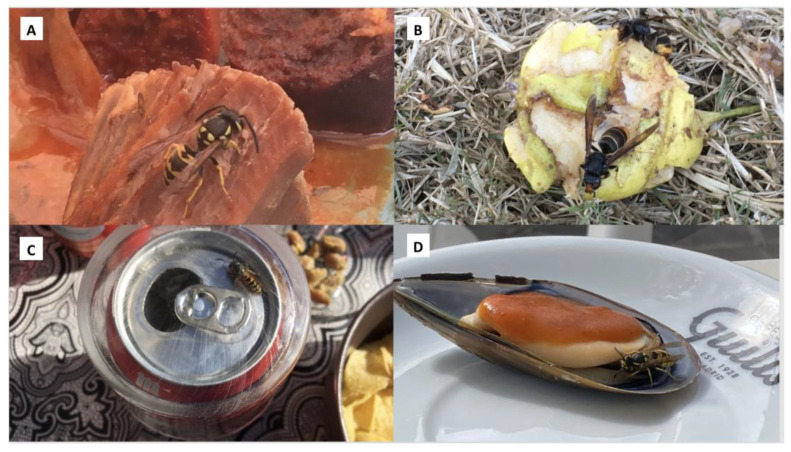
Insects and foods. (**A**) *Vespula* and meat; (**B**) *Vespa velutina* and fruit; (**C**) *Vespula* and beer; (**D**) *Vespula* and shellfish.

The areas where the images were taken were in northern Spain (27 insects), central Spain (25) and southern Spain (19). Most (97.1%) of the images were obtained in the 6 months between June and November, and the other two insects were observed in December and January, respectively. There were no images from the spring. The identified insects were 56 *Vespula* (43 *V. germanica* (Fabricius, 1793), 5 *V. vulgaris* (Linnaeus, 1758) and 8 *Vespula* spp.), 7 *Vespa* (1 *V. crabro* (Linnaeus, 1758), 6 *V. velutina*), 4 *Polistes* (2 *P. dominula* (Christ, 1791), 2 *P. gallicus* (Linnaeus, 1767)), 2 *Cerceris* spp., 1 *Bombus terrestris* (Linnaeus, 1758) and 1 *Apis mellifera* (Linnaeus, 1758). Some examples can be seen in Figure 1. Most of the insects were in restaurants or house terraces (64%). Other places were gardens or the countryside (10), swimming pools (3) and indoors (4).

### 2.2. Foods Related to the Insects

The foods associated with the insects were meat in almost half of the images (47.9%), seafood in 11 (15.5%), sweets in 10 (14%), alcohol beverages in 8 (8.45%), fruits and vegetables in 5 (6%) and others in 3 (4.22%). Table 1 shows the Hymenoptera found on each food. Regarding the type of nutrients, the insects were on carbohydrate-rich foods in 24 cases (8 of them were alcoholic beverages) and proteins in 47. 

No positive correlation was found between the insects and fat-rich foods such as butter, oil, etc. All the insects were found in connection with carbohydrate-rich foods like cakes or alcohol, but only *Vespinae* species were found on protein-rich foods (meat, fish, and seafood). The predictive positive value (PPV) of finding a *Vespula* in a protein-rich food was 89% (*p* < 0.01), and specifically, meat was 91% (*p* < 0.05), while the negative predictive value of it being a *Polistes* was 84% (*p* < 0.05). The different insects, the types of food involved, their predictive value and their statistical significance are shown in Table S1 in the repository. 

The two *Vespa* species were found in north–west Spain. There were no differences concerning *Vespula* and *Polistes* between the north and south. In the central area, all the images obtained were of *Vespula*. 

## 3. Discussion

In this study, we investigate the Hymenoptera found in human food environments to improve insect identification in patients stung while eating or preparing food and, therefore, to improve the diagnosis in allergic patients. 

Not all vespid species have the same foraging habits, and their behaviors can vary widely. This has been reflected in our study, which found *Vespinae*, mainly *Vespula*, as the only Hymenoptera found in connection with human food and almost the only one when the food is protein-rich. The presence of other *Vespidae*, such as *Polistinae* or members of the *Apidae* family, is anecdotal. 

The Spanish *Alergológica* study [10] provides data on the incidence and prevalence of allergic reactions to Hymenoptera stings and the prescription of Hymenoptera venom immunotherapy, showing increased sensitization to *Polistes* compared to *Vespula*. A recent study from our working group [11] has shown the widespread distribution of *P. dominula* in all Spanish provinces, closely followed by *Vespula germanica*, another widespread species. In this study, the distribution maps of vespids were compared with the prescription of venom immunotherapy. The prescription data reflected the inter-regional variability, with the predominance of *Vespula* venom (due to *Vespula* and *Vespa*) in the north and that of *Polistes* in central and southern Spain, although there has been an increase in *Polistes* VIT in both areas in the last 10 years.

In this study, there are hardly any images of *Polistes*, although the two previous studies have established the importance of Polistes venom allergy at a national level. 

We received the pictures of our study mainly during the summer months and early autumn. The degree to which vespids are attracted to human food can vary throughout the year [3]. During the late summer and early fall, when the natural food sources of wasps become scarcer, they may become more aggressive in searching for alternative food sources, including human food. 

Most of the images were taken outdoors, mainly on the terraces of restaurants or private houses. All the insects found indoors were in homes, except one taken in a bar. We found no presence of insects in markets, bakeries or food stores.

The correct identification of the stinging insect is a key point in the diagnosis of Hymenoptera-allergic patients in order to choose the right venom immunotherapy [12,13]. Adults are poor discriminators in distinguishing stinging insects and nests, with the exception of the honeybee. Patients incorrectly identify vespids between 27.7% and 41.9% of times and nests in 70.5% to 81.7% of cases [14]. 

Most of the Hymenoptera that are relevant from an allergology point of view are eusocial. Worker bees and vespids sting in defense of their nest when they perceive that it is threatened, and they also sting in self-defense or to protect their food resources [15]. In the south of Europe, the double sensitization to *Vespula–Polistes* is frequent, more frequent even than the double sensitization to *Apis–Vespula*. In Spain, the double positivity to *Vespula–Polistes* has been found to range from 50.5% to 61.5% in the multiple studies conducted, with most detections being made with sIgE and clinical management being made more difficult by the confusion caused by the cross-reactivity between the allergens from the different vespids [12].

Social vespids differ from most other eusocial species in several ways that affect foraging. Vespid colonies do not store food inside the nest, and foraging is basically for two kinds of foods: animal proteins and carbohydrates [16]. Foraged animal protein provides nutrients for larval growth, whereas carbohydrates serve primarily for energy and thermoregulation. At the individual level, carbohydrates represent quick energy, used for fueling flight and body maintenance. At the colony level, proteins and lipids are more critical as they are essential for larval metamorphosis. So, protein foods have a relatively higher value than carbohydrates [17].

*Vespula germanica* is an eusocial, scavenger and generalist vespid that, in the last century, has invaded forested and urban areas in different countries. In invaded areas, this *Vespinae* represents a problem for a variety of human activities. Efficient foraging is crucial for colony development. *V. germanica* and *V. vulgaris* both share a very flexible foraging behavior, as they prey on other insects, feed on honeydew from aphids and fruit and scavenge on carrion, garbage and food associated with human outdoor activities. Due to the changing abundance of larvae within the colony, protein colony requirements vary throughout a season, peaking at the end of the summer [6,18]. This explains the frequency of images sent for our study in the summer months and early autumn and their absence during the spring. The abundance of larvae in the summer months can also explain the presence of *Vespula*, as the main Hymenoptera found in human food, in connection with protein-rich food, which is essential for larval growth.

In this work, most of the *Vespa* specimens found were *V. velutina*. They were present in urban environments with a foraging behavior similar to that of *Vespula* species. *Vespa velutina* adults predominantly sustain themselves on carbohydrate sources like nectar, larval secretions or ripe fruits while foraging protein sources for the developing brood. This protein comes in the form of a ‘flesh pellet’: a small piece of proteinaceous material that the adult carves out from the protein-rich thorax of a captured arthropod or from carrion. In other parts of Europe, anecdotal information suggests that hornets are opportunistic predators that will exploit any protein source they encounter, including fish or meat markets [19]. *V. velutina* has invaded urban areas, nesting in buildings, and developed a health problem in northern Spain during the last few years [11,20]. In some places, it is the first cause of a Hymenoptera venom allergy [21]. *Vespa crabro* is less of a nuisance, more frequent in rural and forest areas [22] and was rarely attracted by human food in our study.

*P. dominula* appears to be an extraordinarily successful invader of new countries/continents, as documented mostly in comparison to other native species [15]. Polistine vespids prey on other insects and collect nectar for their own energetic demands and for the provision of the brood [23]. The presence of *Polistes* in our study was low, and they were always found in connection with carbohydrate-rich foods like sweet drinks, ice-cream or alcohol. Considering that *Polistes* nationwide distribution has been demonstrated and accounts for 33–48% of the venom immunotherapy prescribed in central and southern Spain [11], the absence of food-related specimens suggests that *Polistes* does not sting in a food environment, contrary to what we observed with *Vespula.* The results may indicate that humans are mainly stung by *Polistes* at nesting sites, which is consistent with the situation in Central Europe [24].

The images collected show vespid foraging behavior. Knowing this, we postulate that it would be of great importance to ask those patients who were stung while eating what kind of food was attracting the insect. If the food was meat, there is a 91% probability that the insect was a *Vespula*.

## 4. Conclusions

*Vespula* was the main hymenopteran associated with food environments in our country (78.87%), and in most of the cases (71%), the food implicated was a source of protein, such as meat or seafood. *Polistes* spp. were rarely present, and they were only found in connection with carbohydrates (alcoholic beverages or sweets) in our study.

## 5. Materials and Method

### 5.1. Working Group

We performed an observational prospective study designed and run by a working group from the Spanish Society of Allergology and Clinical Immunology (SEAIC) Hymenoptera Interest Group, consisting of medical allergologists and an entomologist who specializes in Hymenoptera. Members of the SEAIC were asked, through its website (www.seaic.org) to send images of Hymenoptera occurring in environments where there was human food, giving the date of the image and describing the place and its geolocation data. An email address (vespidos@seaic.org) was made available to store the images, as well as folders for online work (Dropbox^TM^; www.dropbox.com).

### 5.2. Method

The working group assigned a numeric code to each batch of uploaded images. The entomologist identified each insect’s genus and species. Then, a database was created with the following information: the number of images per insect, geographic position, sort of environment (restaurant or bar terraces, homes, gardens, etc.) and type of food involved (fruits, meat, fish, selfish, beverages, etc.).

The images of insects obtained outside food environments were discarded. Each set of images corresponding to the same insect and moment was considered to be just one identification.

### 5.3. Statistical Analysis

Quantitative variables are presented as a median (IQR). Categorical variables are shown as percentages. Bivariate analyses were performed using the χ^2^ test, and the non-parametric Mann–Whitney U test was used for assessing differences between insects. *p* values less than 0.05 were considered statistically significant. All statistical analyses were performed using IBP SPSS Statistics for Windows, Version 20.0 (IBM Corp, Armond, NY, USA).

## Figures and Tables

**Table 1:** Insects and source of food.

Insects	Proteins			Carbohydrates				P/C
	Meat	Fish	Shellfish	Fruits	Vegetables	Sweets	Alcohol	Others
*Vespula*	31	6	3	1	2	6	5	2
*Polistes*						3	1	
*V. crabro*				1				
*V. velutina*	2		2	1			1	
*Apis mellifera*							1	
*Bombus*						1		
*Cerceris*	1							1

P/C = protein/carbohydrate. Human foods where the insects were pictured, divided into protein and carbohydrate-rich food.

## Data Availability

The data presented in this study are available in Supplementary Materials.

## Supplementary Materials

The following supporting information can be downloaded at: https://www.mdpi.com/article/10.3390/toxins15120680/s1, Table S1. Insects and source of food: Positive and Negative Predictive Values.
