# Supplementary material for: Hymenoptera Allergy Diagnosis through Their Presence on Human Food
Source: Toxins (Basel). 2023 Dec 1;15(12):680. doi: 10.3390/toxins15120680 (PMC10748280; doi:10.3390/toxins15120680)
Supplement: Supplementary file 1 [file toxins-15-00680-s001.zip › toxins-2687400-supplementary.pdf]

Table S1. Insects and source of food: Positive and Negative Predictive Values

	Vespula YES	*Vespula* NO	Total			
Carbohydrate YES	14	10	24		PPV	0.58333333
Carbohydrate NO	42	5	47		NPV	0.10638298
	56	15	71		*P*	**0.0046**

	Vespula YES	*Vespula* NO	Total			
Protein YES	42	5	47		PPV	0.89361702
Protein NO	14	10	24		NPV	0.41666667
	56	15	71		*P*	**0.0046**

	Polistes YES	*Polistes* NO	Total			
Carbohydrate YES	4	20	24		PPV	0.16666667
Carbohydrate NO	0	47	47		NPV	1
	4	67	71		*P*	**0.0109**

	Polistes YES	*Polistes* NO	Total			
Protein YES	0	47	47		PPV	0
Protein NO	4	20	24		NPV	0.83333333
	4	67	71		*P*	**0.0109**

	Vespa YES	*Vespa* NO	Total			
Carbohydrate YES	3	21	24		PPV	0.125
Carbohydrate NO	4	43	47		NPV	0.91489362
	7	64	71		*P*	0.6817

	Vespa YES	*Vespa* NO	Total			
Protein YES	4	43	47		PPV	0.08510638
Protein NO	3	21	24		NPV	0.875
	7	64	71		*P*	0.6817

	Apis YES	*Apis* NO	Total			
Carbohydrate YES	1	23	24		PPV	0.04166667
Carbohydrate NO	0	47	47		NPV	1
	1	70	71		*P*	0.338

	Apis YES	*Apis* NO	Total			
Protein YES	0	47	47		PPV	0
Protein NO	1	23	24		NPV	0.95833333
	1	70	71		*P*	0.338

	Bombus YES	*Bombus* NO	Total			
Carbohydrate YES	0	24	24		PPV	0
Carbohydrate NO	1	46	47		NPV	0.9787234
	1	70	71		*P*	1

	Bombus YES	*Bombus* NO	Total			
Protein YES	1	45	46		PPV	0.02173913
Protein NO	0	25	25		NPV	1
	1	70	71		*P*	1

	Others YES	Others NO	Total			
Carbohydrate YES	1	23	24		PPV	0.04166667
Carbohydrate NO	1	46	47		NPV	0.9787234
	2	69	71		*P*	1

	Others YES	Others NO	Total			
Protein YES	1	46	47		PPV	0.0212766
Protein NO	1	23	24		NPV	0.95833333
	2	69	71		*P*	1

	Vespula YES	*Vespula* NO	Total			
Meat YES	31	3	34		PPV	0.91176471
Meat NO	25	12	37		NPV	0.32432432
	56	15	71		*P*	**0.037**

	Vespula YES	*Vespula* NO	Total			
Seafood YES	6	0	6		PPV	1
Seafood NO	50	15	65		NPV	0.23076923
	56	15	71		*P*	0.591

PPV= Positive Predictive Value; PPN= Negative Predictive Value; $P < 0.05$ were considered statistically significant.
